# Collisions and Perceptions of Cyberbullying: Comparison of Intergenerational Experiences

**DOI:** 10.3390/ijerph21091148

**Published:** 2024-08-29

**Authors:** Galina Soldatova, Svetlana Chigarkova, Elena Rasskazova

**Affiliations:** Faculty of Psychology, Lomonosov Moscow State University, 119991 Moscow, Russia; soldatova.galina@gmail.com (G.S.); e.i.rasskazova@gmail.com (E.R.)

**Keywords:** cyberbullying, adolescents, young adults, parents, aggressor, victim, bystander

## Abstract

With regard to negative consequences, cyberbullying is recognized as one of the most traumatic types of cyber aggression. The aim is to study the specific features of adolescents and youth’s cyberbullying experience in the role of an aggressor, victim or bystander, as well as awareness on the part of parents of adolescents. A total of 3395 adolescents, youth and parents filled out specially designed questionnaires. Older adolescents turned out to be at higher risk of cyberbullying. In two-thirds of cases, cyberbullying is related to real-life incidents. Aggressors are motivated by domination and entertainment, primarily employing strategies of social exclusion, harassment and denigration. As victims of cyberbullying, younger adolescents turn to their parents and friends for social support, whereas older adolescents and young adults are more likely turn to their peers. In the role of a bystander, almost half of younger adolescents and about a third of older adolescents and young adults choose the prosocial strategy of protecting a victim. The parents often underestimate the experience of encountering cyberbullying or find it difficult to assess such experience. The identified risk groups and strategies and the lack of parents’ awareness are important to take into account when drawing up cyberbullying prevention programs.

## 1. Introduction

The spread of digital technologies has given rise to new practices of social interactions, which together define digital sociality with its specific forms of behavior, rules and norms [1,2]. Although digital technologies create wide possibilities for social integration, mutual aid and community strengthening activities, the dark side of digital sociality consists of numerous destructive online practices, primarily various types of cyber aggression. With regard to negative personal and social consequences, cyberbullying is recognized as one of the most traumatic types of cyber aggression. Cyberbullying is seen by most authors as an aggressive intentional act carried out by a group or individual, using electronic forms of contact, repeatedly and over time, against a victim who cannot easily defend him or herself [3]. In recent years, this phenomenon has increasingly attracted the attention of scholars and the accumulated empirical data make it possible to deepen the understanding of this destructive practice. The research aim is to study the specific features of adolescents and youth’s cyberbullying experience in the role of an aggressor, victim or bystander, as well as awareness on the part of parents of adolescents. In accordance with the current state of the research field, this paper comprehensively addresses three main lines: (1) to what extent cyberbullying is a product of mixed online/offline reality; (2) what the specifics of the experience of encountering cyberbullying and its perception in different generations are; (3) what the psychological mechanisms and behavioral strategies in different roles of cyberbullying are. Let us focus on several important areas of cyberbullying analysis and set out the relevant research questions.

### 1.1. Cyberbullying and Bullying

Currently, there are three main perspectives in the scientific community on cyberbullying: (1) cyberbullying is a form of face-to-face bullying carried out using digital tools; (2) certain aspects of cyberbullying are similar to traditional bullying, but only under specific circumstances; (3) cyberbullying is a separate and distinct phenomenon [4]. The intention of harm, repetition and power imbalance between a victim and a bully are usually identified as common criteria for face-to-face bullying and cyberbullying [5]. Despite the contradictory approaches, most authors agree on cyberbullying specifics determined by digital sociality (anonymity, infinite audience, limited adult supervision, online disinhibition effect, expansion of spatial and temporal boundaries, replication of damage source and virality, limitless victimization risk, etc.) [5]. In most empirical studies on the prevalence of bullying and cyberbullying among adolescents and youth, the results show a connection between these destructive practices and the overlapping of the said practices, while bullying exclusively in a digital form is not so common [6,7,8]. Thus, it seems important to study the relationship between traditional bullying and cyberbullying, using the modern methods of analyzing social practices within a mixed offline/online reality [1,9]. Given the ongoing digitalization of everyday life, the first research question is: *To what extent is cyberbullying a separate online phenomenon or a part of mixed offline/online reality? (RQ1)*.

### 1.2. Cyberbullying and Age Groups

As another research angle on cyberbullying, the generational aspect should be highlighted. Cyberbullying is most often viewed in relation to school-age students, primarily adolescents, given their age and psychological characteristics and heavy social media use [10,11,12,13]. However, a rather large body of research is devoted to university students [14,15]. The role of age in engaging in cyberbullying is discussed using samples of children, adolescents and youth, although the results are often contradictory [15,16,17]. The second research question is: *Which age group of the digital generation is most at risk of encountering cyberbullying? (RQ2)*.

### 1.3. Aggressors: Behavioral Strategies and Motives

The same as traditional bullying, cyberbullying is characterized by a complex role-based structure that includes a victim, aggressor, bully–victim and bystander who can remain in a passive role or take an active role of a victim defender or bully assistant [18,19,20]. While cyberbullying and face-to-face bullying share a common role-based structure, specific strategies of Internet behavior are identified [21,22].

The most common forms of cyberbullying or aggressor’s behavioral strategies consist of outing and trickery (sharing someone’s secrets or embarrassing information or pictures/videos online, doxxing), denigration (defamation, spreading rumors, humiliating online polls and hate groups), verbal harassment (insults, verbal abuse, threats and harassment), impersonation (assuming another person’s identity online through a fake account or hacking, catfishing) and exclusion (excluding someone from a social media group) [5,23,24,25,26,27]. To understand the nature of perpetrators’ behavior, it is important to analyze their motivation. Studies show that cyberbullies’ motives are a desire to redirect feelings, show one’s negative attitude, take revenge, make oneself feel better, get approval from the reference group, raise or maintain one’s status, boredom, entertainment, dominance and pleasure from harming another [28,29,30,31]. In terms of social relations between the victim and the aggressor, both anonymous users and real acquaintances of different levels of closeness can act as aggressors in a cyberbullying situation [32,33]. The third research question is: *What are the most common motives and behavioral strategies of aggressors in cyberbullying and their social relations with the victim according to different generations? (RQ3)*.

### 1.4. Victims: Prevalence, Emotional Distress, Consequences and Social Support

When analyzing cyberbullying, special attention is paid to the prevalence of victimization. In survey studies, its rates vary significantly from 10 to 40% [34], in some studies reaching more than 70% [35,36]. Studies on the role of COVID-19 in the dynamics of victimization in cyberbullying situations show two opposite trends: increase in prevalence in many Asian countries and Australia and decrease in Western countries [10]. To a large extent, the differences are determined by cultural characteristics and research methods, including the wording of questions, answer options, the measured period of time of an encounter with cyberbullying, etc. [37]. At the same time, scholars are unanimous in understanding the severity of emotional distress caused to victims by incidents of cyberbullying, which may even lead to suicidal thoughts and actions [7,34,38,39,40]. In addition, the intensity of emotional distress may vary depending on the type of cyberbullying and coping strategies of a victim [23,40,41]. Based on the survey studies, problems with life satisfaction, self-esteem, ambitions, communication, sense of security, happiness and optimism, difficulties in school and at home are highlighted among the socio-psychological effects of cyberbullying related to the success of socialization [11,42]. To identify risk groups, the fourth research question is: *At what age are victims most emotionally vulnerable and experience the most significant psychological problems after facing cyberbullying? (RQ4)*.

Considering the age and psychological characteristic of adolescents and youth and one of the key characteristics of bullying and cyberbullying, power imbalances in role-based structures increase the importance of coping strategies such as social support. The main sources of social support are family, friends and school/university staff. A high level of social support is associated with a lower risk of victimization in cyberbullying [34,43]. Social support can be considered as a structural dimension related to the size of a social network and as a functional dimension related to the utility of social support, that is, the opportunity to receive informational, emotional and instrumental support. At the same time, the specifics of digital sociality can complicate access to social support in real life, especially in such a stressful situation as cyberbullying. In this regard, the results of one study show that about half of cyberbullying victims did not inform anyone about the incident [44]. On the one hand, social exclusion is a serious and widespread effect affecting victims of cyberbullying. On the other hand, victims of cyberbullying, especially among adolescents, are those who already have difficulties with communication and socialization. A meta-analysis of research on the subject summarizes data showing that social support can have a significant impact on the victim’s emotional experiences and well-being and help prevent new incidents of cyberbullying [45]. This way, social support as a coping strategy for a victim in a cyberbullying situation deserves special attention. Continuing the previous theme of identifying at-risk groups, the fifth research question is: *What social support resources in the role of victim do certain age groups choose? (RQ5)*.

### 1.5. Bystanders: Behavioral Strategies

While looking into the problem of cyberbullying, researchers and educators’ attention focuses on bystanders, in addition to victims and aggressors [17,46,47,48]. Bystanders represent the most numerous group in a cyberbullying incident and can have a significant impact on its development—lessening or, conversely, worsening its impact [49]. The research shows that the negative effect on cyberbullying victims increases largely due to the growth of the audience of passive bystanders who act as supporters of a perpetrator in the eyes of a victim and thus legitimize and reinforce aggression [50,51]. Bystanders can support aggressor’s actions with reposts and comments, which expands the circle of those involved in cyberbullying and causes significant harm to the victim. However, the transition from a passive bystander to a victim’s defender can greatly change the balance of power and affect the reaction of other bystanders and an aggressor directly [50,52].

There are several approaches to classifying the behavioral strategies of cyberbullying bystanders [53]. In one approach, prosocial (helping, comforting and protecting a victim) and antisocial (joining, assisting and reinforcing a bully) behavior of bystanders are distinguished [54,55]. In a more differentiated approach, three types of bystanders’ behavior are distinguished by adding a passive bystanding role to victim’s defenders and aggressor’s assistants [21,54,55]. In some works, defenders of a victim are also divided based on the principle of indirect and direct intervention [56,57]. Based on the analysis of the motivation and behavior of bystanders in cyberbullying incidents, a classification of five categories was proposed. It included the distant bystander (ignoring), the entertained bystander (observing behavior), the conspiring bystander (intentional encouragement, e.g., helping the aggressor), the unintentional instigating bystander and the active/empowered bystander (reporting the cyberbullying to authorities and interacting with the aggressor directly) [58]. In relation to the strategies identified and in terms of the importance of the bystander role as the most common, the research question is as follows: *Are there differences in the preference for behavioral strategies of cyberbullying bystanders across generations? (RQ6)*.

### 1.6. Parents’ Awareness

When analyzing cyberbullying, researchers’ attention also focuses on parents. Parents’ awareness of their children encountering cyberbullying becomes an important factor in both prevention and coping with the incidents [59]. At the same time, research shows that parents are often insufficiently aware of their children facing online risks, including cyberbullying [60,61]. Parents can overestimate encounters with some online risks, and they are more concerned about risks such as negative content and underestimate communication risks that are more important for children and adolescents [62,63]. This situation is often aggravated by the digital divide, when parents, due to their user activity and digital competence, cannot fully act as experts and assistants in the process of digital socialization of their children, leaving them alone with these problems [64,65]. In this regard, our research question is: *What aspects of cyberbullying are underestimated by parents of adolescents? (RQ7)*.

### 1.7. Current Study

The current study is one of the first social–psychological studies of cyber aggression and cyberbullying with a representative sample of three generations and covering the main regions of Russia. Some of the results have already been published [66,67,68,69]. Considering cyberbullying through the prism of three generations (adolescents, youth, parents of adolescents) allows us to implement a holistic approach to this complex problem. It is also important to examine the experiences and perceptions of different age groups in relation to all roles of cyberbullying, highlighting socio-psychological mechanisms and behavioral strategies. The results obtained will help to clarify a number of aspects of digital sociality related to destructive practices and become a point of reference for further research. Data from the Russian survey allow for comparisons with results in other countries and highlight common trends.

## 2. Materials and Methods

### 2.1. Procedures

The survey was conducted in 2018 on multi-stage stratified representative samples of adolescents aged 12 to 17, parents with children aged 12 to 17 and youth aged 18 to 35. All the respondents lived in Russian cities with a population of 100,000 or more. For the study, 20 cities located in 8 Russian federal districts were selected: Southern (Rostov-on-Don, Volgograd), Volga (Kazan, Kirov), Siberian (Kemerovo, Novosibirsk), Far East (Magadan, Petropavlovsk–Kamchatsky, Khabarovsk), North Caucasian (Makhachkala, Vladikavkaz), Northwestern (St. Petersburg, Vologda), Central (Moscow, cities of the Moscow region) and Ural (Tyumen, Yekaterinburg).

The survey was conducted in public places. In each city, several survey locations were randomly selected and at each one, no more than 5–8 adolescents, 5–8 parents and 5 young people were surveyed. Applying quotas to the adolescent and youth gender and age, the study respondents were selected. Applying quotas to the gender and age of their child, the parents were also selected. The survey of both the adolescents and their parents was conducted only if the adolescents used the Internet. The youths were interviewed only if they used the Internet.

The survey was conducted using a personal interview based on questionnaires for each age group. A university network was used to select interviewers with the appropriate professional level to conduct the study. To conduct the survey, 68 experienced interviewer psychologists were chosen. The work of the interviewers was supervised by the staff of the Faculty of Psychology at Lomonosov Moscow State University.

### 2.2. Participants

The study involved 3395 people: 1105 parents of adolescents aged 12 to 17, two groups of adolescents—525 younger adolescents aged 12 to 13 and 1029 older adolescents aged 14 to 17—and 736 young people aged 18 to 35 (see Table 1). Among parents, the adult most actively involved in the adolescent’s life was sampled. Therefore, mothers prevailed among the parents; the rest of the samples were distributed almost evenly by gender. The respondents lived in 8 Russian federal districts. The samples of the adolescents, parents of the adolescents and youth were distributed equally among the federal districts (N = 322), except for the Central District, in which more than the established norm was collected (N = 1141).

### 2.3. Measures

The study was conducted on the basis of specially designed socio-psychological questionnaires. Due to the lack of questionnaires validated on the Russian sample, the questions were developed by the research team on the basis of theoretical and empirical works on the topic and were expertly evaluated by psychologists working in the field of aggression and digital socialization. Appropriate forms of questionnaires were prepared for 4 age groups: adolescents aged 12 to 13, adolescents aged 14 to 17 and parents of the adolescents of these age groups and youth. The questionnaires included several blocks of questions, as well as methods of psychodiagnostics and methodological techniques. The questionnaire for the younger adolescents consisted of 47 questions, for the older adolescents, 65 questions; for the youth, 70 questions; and for the parents, 65 questions. All the questionnaires included similar blocks of questions aimed at studying various aspects of Internet use, impact of online risks and cyber aggression. The article partly presents the results of the survey on experiencing cyberbullying.

#### 2.3.1. Experiencing Cyberbullying

To assess bullying locations, the respondents who experienced cyberbullying situations answered the question “Where did the situations occur?” with three possible answers: “Only on the Internet”, “Mainly on the Internet and sometimes in direct face-to-face communication” and “Mainly in face-to-face communication and sometimes on the Internet”.

To assess cyberbullying experience, the adolescents, youth and parents read the description of a situation and answered the question “It happens that children, adolescents and also adults say or do some offensive things to another person on the Internet and this can happen in various ways over a long period of time. Such behavior can manifest as the following: teasing, calling someone names, making an individual uncomfortable by mocking, harassing, excluding from the general activities of a class or group. This behavior is called cyberbullying. Have you ever encountered the behavior?” (answer options: “Yes”, “No” and “I find it difficult to answer”).

#### 2.3.2. Aggressors

To determine the nature of the aggressor’s social relations with the victim, the respondents answered the question “Who performed actions towards the affected person?” with multiple answer options: “Classmates”, “Students from the same year level”, “High school students”, “Teachers”, “Acquaintances”, “Internet friends”, “Parents”, “Siblings”, “Friends” and “Anonymous users”. The parents were also provided with an option to answer “I find it difficult to answer”.

To study the perceptions of aggressors’ motivation in cyberbullying situations, the older adolescents, youth and parents answered the question “Why do you think a person bullies another one on the Internet?” (for the parents, the wording was “Why do you think adolescents get involved in cyberbullying?”) with multiple answer options: “Experiment and look at the reaction of other people”, “Just to have fun”, “Accompany their friends”, “Express one’s attitude”, “Release accumulated negativity”, “Show one’s strength and superiority”, “Achieve one’s own goal or personal benefits”, “Maintain one’s reputation”, “Take revenge” and “Harm another person”.

To determine the strategies of aggressors’ behavior in a cyberbullying situation, the older adolescents, youth and parents answered the question “When you were involved in or observed a cyberbullying situation, what actions were performed towards the affected person?” with multiple answer options: “Was removed from the friend list”, “Was excluded and removed from a group chat or community”, “Personal data, photos and videos from the personal page were used against the person”, “Personal data (first name, last name, photos, etc.) from the online profile was used to create a fake account”, “False information about the person was posted”, “The account password was stolen in order to post or send negative and inappropriate information on the person’s behalf”, “Rude and unpleasant polls about the person were created”, “Groups, communities or pages on social networks were created, where offensive information about the person was posted” and “Insulting and humiliating content about the person was sent to the friends, parents and teachers”.

#### 2.3.3. Victims

In order to identify victims of cyberbullying and the level of emotional distress, the older adolescents and young people were asked the question “When actions related to cyberbullying were taken against you, how upset were you?”. For the parents, the following wording was used: “When actions related to cyberbullying were taken against your child, how upset was your child?”. The rating scale consisted of 5 response options: “Extremely upset”, “Very upset”, “Slightly upset”, “Not at all” and “I find it difficult to answer”.

Afterwards, the older adolescents and youth who recognized themselves as victims of cyberbullying and the parents whose children were cyberbullied answered the single choice question about the duration of the experience: “How fast did you cope with your distress?” (for the parents, the wording was “How fast did your child cope with the distress?”): “Almost immediately”, “In a few days”, “In a few weeks”, “In a couple of months and even longer” and “I find it difficult to answer”).

All the respondents answered the question about seeking social support in a cyberbullying situation—“Who did you turn to for support in this situation?”—with multiple answer options: “Parents”, “Sibling”, “Friend”, “Teacher”, “Law enforcement agencies (police)”, “Specialized services (psychologist, social worker, etc.)”, “Trusted adult” and “No one”.

The older adolescents and youth who were victims of cyberbullying assessed the impact of the experience on various aspects of their socialization. They were asked to evaluate how much the situation affected their self-esteem, communication, optimism, school/work, life at home, ambitions, reputation according to a 5-point rating scale (0—did not affect in any way, 5—affected very strongly) and two parameters: positive impact or negative impact.

#### 2.3.4. Bystanders

To assess the behavior strategies of a cyberbullying bystander, the respondents were asked the question “When you witnessed the actions, what did you do?” (for the parents, the following wording was used: “When your child witnessed the actions, what did he or she do?”) with multiple answer options: “Supported the user who was targeted”, “Did not intervene because was sure that the others would support the user”, “Did not intervene because did not know the person”, “Supported the user who started bullying”, “Did not intervene because had doubts about the person not deserving to be bullied”, “Left the page, resource, or community”, “Told adults about the situation”, “Did not do anything” and “I find it difficult to answer”.

### 2.4. Data Analyses

Data processing was carried out with SPSS 23.0 based on descriptive statistics and methods of group comparisons (chi-square test, ANOVA, *t*-test).

## 3. Results

### 3.1. The Ratio of Bullying to Cyberbullying

**Figure 1:**
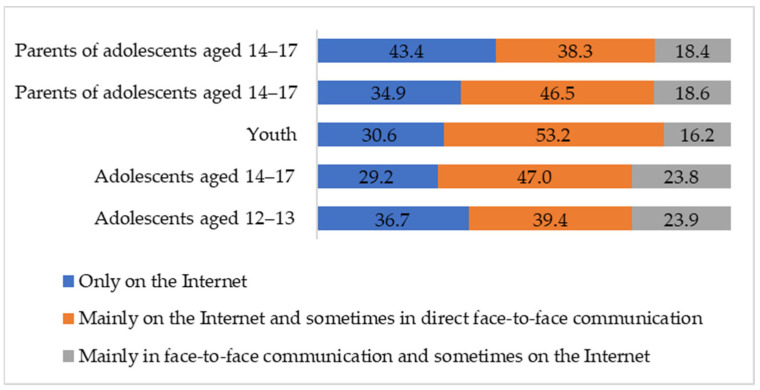
Where bullying incidents occur according to adolescents, youth and parents, by percentage.

To assess the ration of bullying to cyberbullying, participants were asked where the bullying incidents typically occur to their mind. Chi-square tests were used to reveal the differences between 5 groups of respondents. In about a third of the incidents, cyberbullying situations are limited exclusively to the digital space. However, for two thirds of adolescents and 70% of young people, cyberbullying is a part of bullying (Figure 1). For every fourth adolescent, bullying occurs in real space; such a scenario is less common among young people and parents, but in general, the differences between the groups are small (χ^2^ = 26.68, *p* < 0.01, Cramer’s V = 0.10). The combination of bullying and cyberbullying with a preponderance of online space turned out to be the most common situation for the adolescents, young people and parents of the younger adolescents.

### 3.2. Encountering Cyberbullying Situations: Age Characteristics

**Figure 2:**
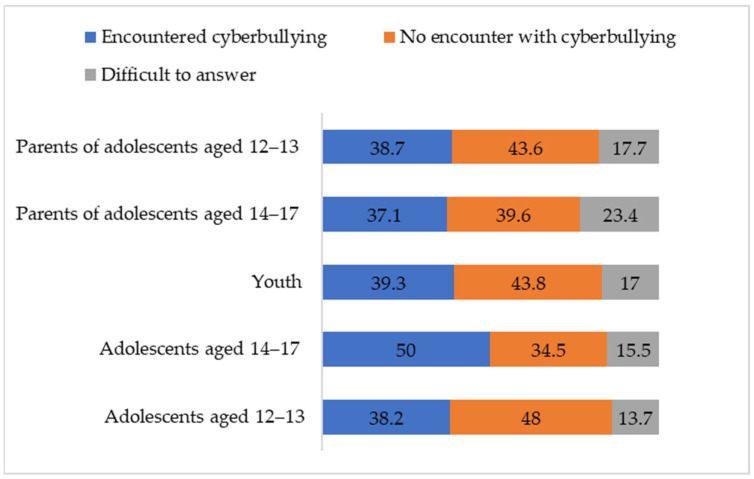
Encountering cyberbullying situations by percentage.

Chi-square tests were used to compare the experience of cyberbullying between age groups (adolescents aged 12–13, adolescents aged 14–17 and youth) and between adolescents and parents (separately for 12–13 years old and 14–17 years old). On average, more than a third of the respondents are familiar with such situations and among the older adolescents, half of them (see Figure 2). The older adolescents encounter cyberbullying situations more often than younger ones and youth, but the differences between the groups are small in size (χ^2^ = 35.44, *p* < 0.01, Cramer’s V = 0.09). Parents of adolescents aged 14–17 less frequently report that their children encountered cyberbullying situations than adolescents aged 14–17 (χ^2^ = 14.05, *p* < 0.01, Cramer’s V = 0.10) while parents of adolescents aged 12–13 are fairly accurate in their appraisals.

### 3.3. Aggressors in a Cyberbullying Situation

#### 3.3.1. The Nature of the Aggressors’ Social Relations with a Victim of Cyberbullying

Frequencies of the aggressors’ social relations with a victim of cyberbullying are presented in Table 2. Chi-square tests are used to compare frequencies among five groups—adolescents aged 12–13, adolescents aged 14–17, youth, parents of adolescents aged 12–13 and 14–17.

Only one third of adolescents and youth respond that cyberbullying initiators are anonymous users. The most frequent aggressors on the Internet are, with different degrees of proximity, in the system of social ties. These are usually classmates (68% among adolescents), students from the same year level or friends, although, for the older adolescents and youth, the role of acquaintances (37% and 39%, respectively) is increased, including online ones (24% and 33%). The role of high school students is indicated by every fifth adolescent, but still it is not as great as one might expect. Typically, teachers, parents and siblings are far from being aggressors in the digital world.

Compared with the adolescents, the youth and parents were less likely to mention classmates (46% on average) and students from the same year level and high school students as bullies; in the other instances, the differences in assessment compared with the adolescents are less pronounced. Almost every third parent of an adolescent aged 14 to 17 considers anonymous users or Internet friends to be cyberbullying initiators, which essentially corresponds to the assessments given by the adolescents of the same age. Nevertheless, the parents often find it difficult to answer this question, especially the parents of the younger adolescents (every fourth parent).

#### 3.3.2. Motivations for Cyberbullying

**Figure 3:**
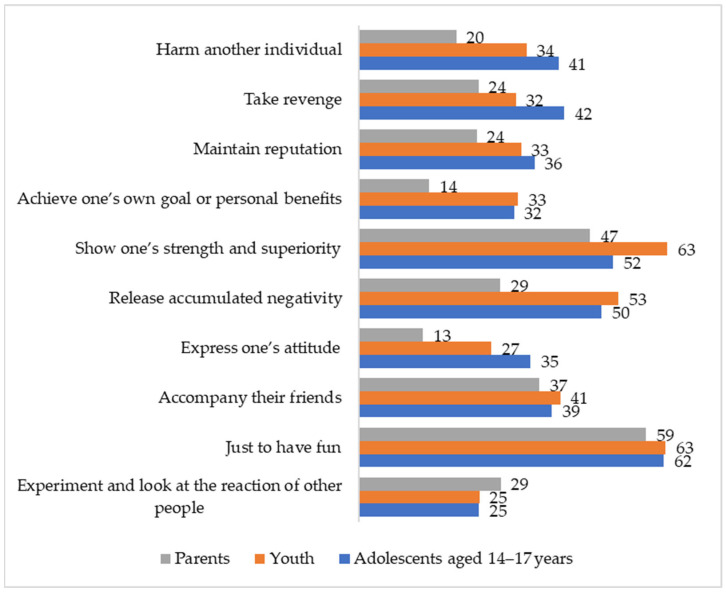
Motivations for cyberbullying according to the older adolescents, youth and parents, by percentage.

More than half of the members of all the generations identified two common motives of aggressors in cyberbullying situations: demonstration of superiority and entertainment (Figure 3). For every second young person and adolescent, another motive is typical, that is, releasing accumulated negativity. The adolescents also often talk about seeking revenge (42%) and harming another person (41%). Approximately the same number of the respondents believe that aggressors do it just because their friends do it—41% of the youth and 39% of the adolescents. A third of the adolescents and youth believe that cyberbullying arises from a desire to maintain a reputation and achieve a certain goal or benefit. A quarter of the adolescents and youth indicate a desire to experiment and see the reaction of other people. A third of the adolescents and a quarter of the youth assume that cyberbullying can be used to express an attitude or one’s own opinion.

Chi-square tests were used to reveal differences in motivation between groups of adolescents 14–17 years old, youth and parents. Parents more frequently consider experiments (χ^2^ = 6.71, *p* < 0.05, Cramer’s V = 0.05) and less frequently consider expression of one’s attitude (χ^2^ = 139.70, *p* < 0.01, Cramer’s V = 0.22), release of accumulated negativity (χ^2^ = 136.25, *p* < 0.01, Cramer’s V = 0.22), demonstration of superiority (χ^2^ = 44.62, *p* < 0.01, Cramer’s V = 0.13), looking for benefits (χ^2^ = 111.42, *p* < 0.01, Cramer’s V = 0.20), maintaining of reputation (χ^2^ = 37.44, *p* < 0.01, Cramer’s V = 0.12), revenge (χ^2^ = 72.92, *p* < 0.01, Cramer’s V = 0.16) and harming (χ^2^ = 110.00, *p* < 0.01, Cramer’s V = 0.20) as a motivation for cyberbullying than adolescents and youth.

#### 3.3.3. Strategies of the Cyberbullies’ Behavior

Frequencies of different variants of cyberbullying behavior are presented in Table 3 as well as chi-square tests comparing differences between 5 groups of participants (adolescents 12–13 and 14–17 years old, youth, parents of adolescents 12–13 and 14–17 years old). Every second adolescent and young person indicates three widespread behavioral strategies aimed at a victim: excluding from a group/community/friend list; bullying using data posted on a personal page; and posting false information about the target. One in three adolescents and one in four young people encountered insulting polls about the victim. A third of the young people and a quarter of the older adolescents indicate the creation of hate groups as aggressive acts. One in four young persons and one in five adolescents report a form of cyberbullying when fake accounts are created on behalf of the victim. A fifth of the young people and a sixth of the adolescents talk about sending humiliating and insulting information about the victim to his or her relatives and acquaintances. The rarest aggressive act in a cyberbullying situation is stealing the password from the victim’s account and sending negative information on her or his behalf. It is worth noting the differences in the responses of the parents, who often underestimate what aggressive acts cyberbullying victims can be subjected to.

### 3.4. Cyberbullying Victims

#### 3.4.1. Prevalence of Victimization

Below, only the answers received from those respondents who admitted to being cyberbullying victims are analyzed. When the parents were interviewed, only those whose children were victims provided answers. Almost every second older adolescent (54.7%) and every third young person (39%) were cyberbullying victims. At the same time, among the parents of the younger and older adolescents, only a third were aware of their children encountering such experience (31.9% and 33.2%).

#### 3.4.2. The Strength and Duration of the Victim’s Emotional Response

**Figure 4:**
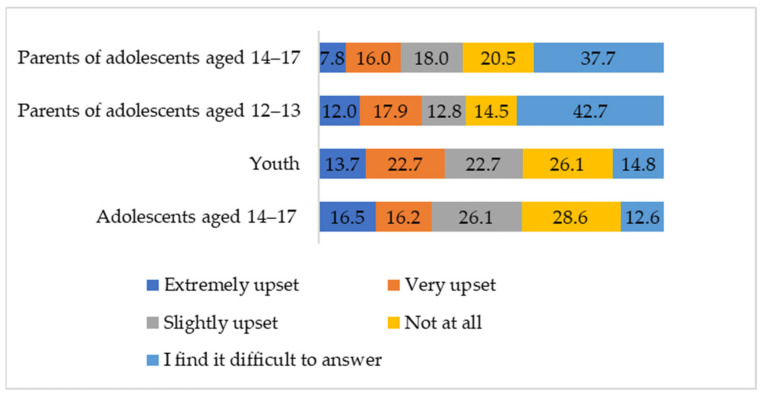
The degree of the emotional reaction to cyberbullying (according to the assessment of the last such situation that the individual encountered), by percentage.

A chi-square test was used to reveal differences in replies about emotional reaction to cyberbullying and its duration between 4 groups. More than a third of the youth and adolescents (41%) believe that the last cyberbullying situation they were in seriously or very seriously affected them emotionally, and only one in four states that cyberbullying does not affect them in any way (Figure 4). Among the older adolescents, the emotional reaction is somewhat stronger, even though the scores are close to the assessment given by the young people, whereas the parents often find it difficult to answer the question about the emotional reaction of their children (χ^2^ = 118.16, *p* < 0.01, Cramer’s V = 0.18).

**Figure 5:**
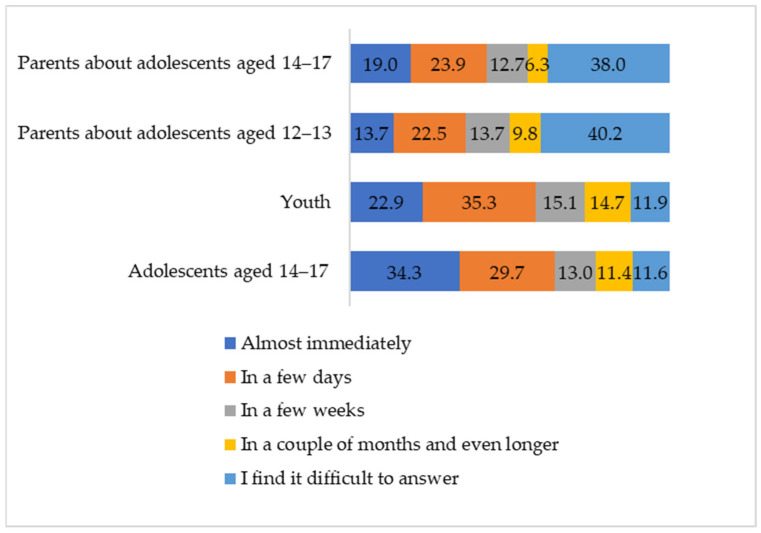
The duration of the emotional reaction to cyberbullying (according to the assessment of the last such situation that the individual encountered), by percentage.

For every fourth adolescent who experienced cyberbullying, it took several weeks or longer to recover; and among the youth, the scores are even higher (χ^2^ = 116.09, *p* < 0.01, Cramer’s V = 0.20). For at least one in ten young people, it took more than two months or longer (Figure 5). A third of the adolescents and one in five young people stated that they immediately rid themselves of emotional distress. For about a third of them, it took a short period of time. Just as with the degree of the emotional reaction, the parents of the adolescents who experienced cyberbullying often find it difficult to answer how long their children were in distress and the level of it.

#### 3.4.3. Seeking Social Support

Frequencies of seeking support and help in the situations of cyberbullying are presented in Table 4 as well as comparisons of 5 groups (adolescents 12–13 and 14–17 years old, youth, parents of adolescents 12–13 and 14–17 years old). While for the adolescents aged 12 to 13, parents and friends are equally important for overcoming distress related to cyberbullying, for the adolescents aged 14 to 17, parents and youth recede into the background. Every fourth adolescent aged 14 to 17 does not turn to anyone, and among the young people, one in three copes with the situation on their own. One adolescent out of five or six turns to siblings. Among the adolescents aged 12 to 13, one in ten reports getting help from a teacher, but starting from the age of 14, reaching out to a teacher becomes a rare occasion. Law enforcement agencies and specialized services are rarely considered as sources of support.

The parents who are aware of the cyberbullying situation happening to their children overestimate how often the adolescents turn to them for help and greatly underestimate how often the adolescents turn to their friends or do not look for help at all.

#### 3.4.4. The Consequences of Cyberbullying

**Figure 6:**
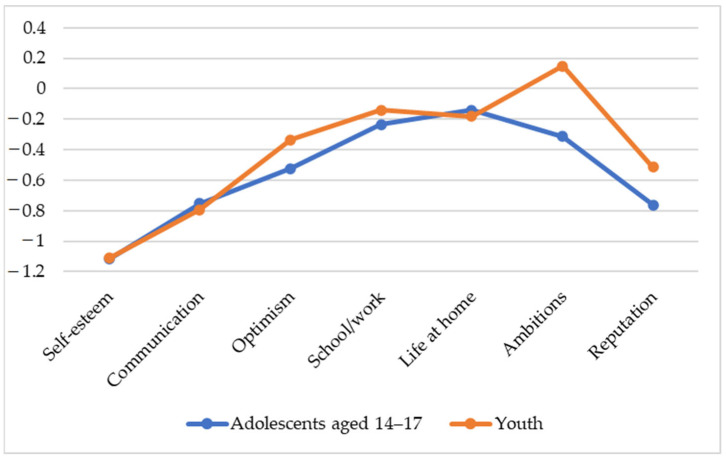
The impact of cyberbullying on various aspects of the lives of the adolescents and youth, scores.

The older adolescents and youth additionally assessed the extent to which the cyberbullying situation negatively or positively affected their self-esteem, communication, optimism, school/work, life at home, ambitions and reputation (Figure 6). We used *t*-tests to reveal differences between adolescents and youth, and analysis of variance with repeated measurements (2 groups × 7 domains) was used to reveal differences in the subjective impact of cyberbullying on different domains. The adolescents and young people are almost equally in agreement that cyberbullying has the most negative impact on self-esteem, communication and reputation and to a lesser extent on optimism.

Cyberbullying has practically no effect on school/work, life at home and ambitions. At the same time, the adolescents and youth totally disagree about its influence on ambitions; the adolescents believe that cyberbullying negatively affects ambitions, the young people, on the other hand, believe that it positively affects ambitions (*t* = −2.49, *p* < 0.05, r = 0.09).

According to ANOVA with repeated measures, the negative impact on self-esteem, communication, reputation is more pronounced than on optimism, school/work, life at home, ambitions (F = 29.97, *p* < 0.01, η^2^ = 0.05), while the difference between the adolescents and youth is not revealed.

### 3.5. Cyberbullying Bystanders

**Figure 7:**
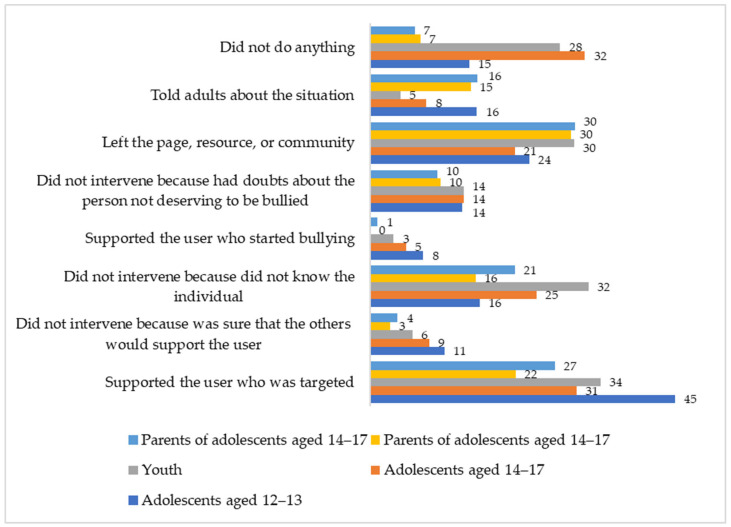
Bystanders’ behavior strategies in a cyberbullying situation, by percentage.

Differences in behavioral strategies of cyberbullying bystanders between 5 groups of participants (adolescents 12–13 years old, 14–17 years old, youth, parents of adolescents 12–13 years old and 14–17 years old) were revealed using Chi-square tests. Every third adolescent and young person and adolescents aged 12 to 13 even more often are ready to support a victim in a cyberbullying incident (Figure 7). However, since the age of 14, inaction becomes an increasingly common reaction and one in five leaves the page, resource or community where the incident happened. Every fourth older adolescent and young person does not intervene because they do not know the victim. Every eighth individual does not intervene because they are not sure whether the victim does not deserve what is happening to him or her. However, among the adolescents aged 12 to 13, not only the number of children who support the victim is higher, but also the number of those who support the aggressor—about one out of thirteen children acts this way.

With age, people are more likely to be inactive; they do not interfere because they do not know the victim (χ^2^ = 15.83–20.38, *p* < 0.01, Cramer’s V = 0.13–0.15). They leave the resource where they encountered cyberbullying (χ^2^ = 7.73, *p* < 0.05, Cramer’s V = 0.09) and less often support the victim or turn to adults (χ^2^ = 12.89–18.58, *p* < 0.01, Cramer’s V = 0.12–0.14).

The parents generally overestimate what their children do when they encounter cyberbullying, that is, leave the online resource. At the same time, their answers about their children sharing with adults about the incident and not intervening because they do not know the target are quite accurate. The parents underestimate both inaction and support for the victim, and almost no one knows about the adolescents who supported the aggressor. A significant percentage of the parents (20.4% of parents of adolescents aged 12 to 13 and 26.9% of parents of adolescents aged 14 to 17) find it difficult to answer this question.

## 4. Discussions

### 4.1. The Prevalence of Cyberbullying and Its Correlation with Bullying

In line with *RQ1 To what extent is cyberbullying a separate online phenomenon or a part of mixed offline/online reality*, the results show that cyberbullying unrelated to any actions performed in the real world can be seen only in about a third of incidents. This is consistent with both the methods of modern research, which demonstrate the increasing penetration of online and offline spaces, and cyberbullying empirical studies that demonstrate the cyberbullying connection to face-to-face bullying [6,7,8,19]. In this regard, cyberbullying is a phenomenon of the mixed reality that suggests the convergence of virtual and real life.

Cyberbullying turns out to be a common risk faced by a third to half of Russian adolescents and youth. This is generally consistent with research data in other countries [35,36] and with the data from a study of Russian adolescents who encountered cyberbullying in the form of hate groups [66]. According to *RQ2 Which age group of the digital generation is most at risk of encountering cyberbullying*, older adolescents turned out to be this group. Despite the inconsistency of data on the relationship between age and cyberbullying [15,16,17], the results obtained are fully explained by a combination of several factors. High user activity of older adolescents—who are ahead of younger adolescents in this parameter and are catching up with young people [63,70]—and their willingness to explore new online communication spaces, in combination with a highly expressed need to communicate with peers, increase the risk of encountering cyberbullying.

### 4.2. Cyberbullies

In this section, we discuss the results obtained in response to *RQ3 What are the most common motives and behavioral strategies of aggressors in cyberbullying and their social relations with the victim according to different generations*.

#### 4.2.1. The Nature of the Aggressors’ Social Relative to the Victim

A social circle in real life (classmates, students from the same year level, acquaintances) acts as the main source of cyberbullying, which also proves the connection between traditional bullying and cyberbullying, especially for schoolchildren [32,33]. Nevertheless, up to a third of the adolescents and youth report that the aggressors were anonymous users. Anonymity as a specific feature of digital sociality plays an important role in the start of cyberbullying. The ability to remain anonymous increases the number of incidents due to the feeling of impunity to perform aggressive actions in the online space, a lack of understanding the severity of emotional distress of the victim and the online disinhibition effect [5,71,72]. Building up online social capital through weak connections—online pen pals—turns out to be a potential source of victimization risk, which is relevant for older adolescents and especially young people who actively expand their network [73,74]. With age, the number of aggressors increases among Internet friends.

#### 4.2.2. The Aggressor’s Motives

All generations believe that the most common motives of cyberbullies are dominance and entertainment. According to the quadripartite violence typology [75], the demonstration of strength and superiority, as well as maintaining a reputation, which was mentioned by a third of the respondents, can be attributed to controlled–appetitive aggression or reward aggression [76]. The choice of cyberbullying as a behavioral strategy can then be based on the need of adolescents and young people to increase or promote their social standing in the peer group [31]. This is also reflected in the choice of the motive of “accompanying friends” that demonstrates both the group nature of cyberbullying and the use of cyberbullying to fit into the social group and to satisfy the basic need for belonging in such a destructive way. While dominance is also a key motivation for traditional bullying, entertainment is a part of impulsive–appetitive or recreational aggression and is more common in cyberbullying [76,77]. The specifics of digital sociality contribute to this: the anonymity of aggressors or them being able to distance themselves, the lack of social cues or a direct emotional reaction from the victim and undermining the damage caused by the aggressor result in most of the respondents unanimously highlighting this motive. The specifics of digital sociality that determine destructive online behavior can also explain the common motivation of releasing accumulated negativity, that is, redirecting feelings [77]. The Internet is becoming a place to “take out” negative experiences and emotions, mainly by redirecting these emotions onto others who are weaker and more vulnerable (the phenomenon of scapegoating). Revenge is regarded as reactive aggression and, as other studies show, is one of the most common motives of cyberbullies [31,77]. This indicates a low level of conflict competence among the younger generation, when, in a conflict, destructive behavioral strategies aimed at humiliating another person are preferred rather than effective ways to resolve disagreements and clarify the situation in face-to-face communication. Intentional harm to another—recognized as a motive by more than a third of the adolescents and young people—might be consistent with the data on the development of the dark triad of personality among cyberbullies [78,79] and consequently the lack of remorse in such behavior.

#### 4.2.3. The Aggressor’s Behavioral Strategies

Among the most common cyberbullying strategies, adolescents and young people identify social exclusion, harassment by using information from the victim’s personal page and denigration (spreading rumors). Such results are consistent with the proposed classifications of cyberbullying tactics and types [5,23,24,25,26,27]. Spreading rumors and exclusion are attributed to relational bullying and appear to be the most common types of face-to-face bullying [33]. Exclusion in social media is associated with consequences such as depression, anxiety, social disadaptation and suicide risk [80]. Meta-analyses show that exclusion as a form of cyberbullying also has consequences at the neurobiological level, leading to increases in deviance and stimulus detection responses, as well as in emotional attention and emotional regulation [81]. Developmental needs include high-quality friendships, peer acceptance and close relationships with non-familial adults as well as dynamic cognitive, social, personal and emotional needs [82]. Self-determination theory contends that individuals have basic psychological needs for autonomy, competence and relatedness [83], including high-quality friendships, peer acceptance and close relationships for adolescents and young adults. Social ostracism deprives an individual of a sense of belonging that is a component of the well-being of adolescents and youth [84]. Exclusion also makes it impossible for victims to seek social support—one of the constructive coping strategies in a cyberbullying incident.

As a specific feature of digital sociality, the tactics of cyberbullies to use information that victims post on their personal pages on social networks can be highlighted. The desire to share information about yourself while working on self-presentation is a common practice in social media, especially for such active users as adolescents and youth. However, the changing boundaries of privacy and the lack of competence in handling personal data online become an area of concern in relation to potential harm to children and young people [85,86]. The need for self-presentation on social media can take the form of risky behaviors such as self-disclosure and oversharing online [87,88,89]. According to the data of a Canadian study, on the one hand, adolescents can express their privacy concerns and at the same time demonstrate the opposite behavior on social networks; on the other hand, adolescents, especially older ones, believe that they have “nothing to hide” online and therefore do not consider privacy to be relevant for them [90]. Thus, the specifics of self-presentation and contradictive privacy management online can lead to an increased risk of victimization in a cyberbullying incident.

Other cyberbullying tactics (creating fake accounts on behalf of the victim, hate groups, hacking the victim’s account, etc.) that are more resource-consuming or require digital competence are less common. Thus, the easier it is to perform aggressive actions against the victim, the more often they occur.

### 4.3. Victims

According to this study, slightly fewer than half of the adolescents and more than a third of the young people reported experiencing victimization in a cyberbullying situation, which is consistent with other studies, causes angst and confirms the importance of the problem [34,35,36].

#### 4.3.1. Emotional Impact and Consequences for Victims of Cyberbullying

According to the answer to *RQ4 At what age are victims most emotionally vulnerable and experience the most significant psychological problems after facing cyberbullying*, the importance of the problem is also supported by the data on the strength and duration of the victims’ response to cyberbullying. Cyberbullying can be attributed to serious stressful situations that cause emotional distress, feelings of anger, fear and shame, anxiety-depressive experiences, suicidal thoughts and degrade performance [23,40,41].

The victims among the older adolescents and young adults highlight a negative impact on self-esteem, communication and reputation—that is, on the most important factors determining the success of socialization—as the most prominent consequences of cyberbullying. This correlates with the results described above about the most common relational bullying in the form of social exclusion, spreading rumors and harassment on the Internet. Such data are consistent with other studies [11,42]. The impact of victimization on ambition has been poorly studied. The results show that although the effects of cyberbullying on ambition, according to the victims, did not turn out to be so strong, nevertheless, when comparing the two age groups, it turned out that the adolescents believe that cyberbullying negatively affects ambitions and the young people believe that it has a positive effect. It is possible that in the short term, cyberbullying makes people less ambitious while undermining their self-esteem and reducing performance, but in the long term, it can stimulate their desire to overcome a traumatic experience and prove their capabilities and abilities, which youth might have already learnt from their own experience as they were growing up.

#### 4.3.2. The Specifics of Seeking Social Support

In accordance with *RQ5 What social support resources in the role of victim do certain age groups choose*, a risk group—which includes a quarter of the younger and older adolescents and a third of the youth—can be identified. Social support is the most important constructive coping strategy in a cyberbullying situation [43]. Moreover, based on the results of a meta-analysis, social support and connecting with other people act as key protective factors in suicidal behavior, which is the most serious risk for victims of cyberbullying [91]. Thus, a significant number of cyberbullying victims find themselves alone with their trauma, which can enhance and expand its effects on different aspects of life. However, most victims still seek social support. The younger adolescents are more likely to turn to their parents and friends, whereas almost every second older adolescent and a quarter of the young adults turn to their peers, which corresponds to age-related needs for autonomy. Research shows that peer and family support contributes to the development of emotional regulation and decrease in impulsivity in case of cyber victimization, which helps victims better cope with incidents [92]. Although looking for support from teachers is not too common, the younger adolescents turn to them more often than the older ones, which is important to take into account in the development of effective anti-bullying programs [93]. Teachers can play an important role in reducing both bullying and cyberbullying [94] and school and university climate (the level of trust in teachers and school administration, communication norms, digital culture, spoken and unspoken guidelines about bullying and cyberbullying) is one of the environmental predictors of cyberbullying [15,49,95].

### 4.4. Bystanders

Despite the large number and importance of bystanders in the role-based structure of cyberbullying, researchers are just beginning to pay attention to them. Cyberbullying incidents themselves can have a negative impact on bystanders, normalizing attitudes towards violence, blurring personal responsibility, forming moral disengagement and reducing empathy [17,49,96]. At the same time, the actions of bystanders can strongly influence the balance of power in a cyberbullying incident—lessening or, conversely, worsening its effect [52]. In response to *RQ6 Are there differences in the preference for behavioral strategies of cyberbullying bystanders across generations*, we received the following results. Almost half of the younger adolescents and about a third of the older adolescents and young adults choose the prosocial strategy of protecting a victim. The younger adolescents are also more likely to tell adults about the incident, which can help to more effectively end cyberbullying and provide the victim with the necessary social support. Compared to the other age groups, the younger adolescents are not only more often help the victim, but also take the role of reinforcers of the aggressor. This may be due to a lower level of emotional regulation and communicative competence—including in relation to the specifics of the digital environment—which is typical for this age. The younger adolescents, both potential defenders and aggressors’ assistants, turn out to be an important age group that should be given great attention in order to prevent cyberbullying.

With increasing age, avoidance and inaction become more common strategies. This is consistent with the data from other studies [52,97]. Such a decline in the proactive position might be associated with the effect of desensitization that reduces the likelihood of actively engaging in a cyberbullying situation [17,98]. As immersion in the online space increases, a decrease in sensitivity to cyberbullying situations can be seen as a negative effect of digital sociality. As other studies also show, the most common reason for passive behavior and inaction is unacquaintance or slight acquaintance of the victim [21,54]. Inaction due to blurring responsibility (“others will protect”) can be explained by Genovese syndrome and the bystander intervention model [99], which is actively used to analyze cyber aggression situations [100]. This model also explains the choice of inaction by perceived fairness (“he/she deserved it”), which leads to the victim blaming that is often seen in cyberbullying [101]. It can be assumed that the specifics of digital sociality such as synchronous/asynchronous communication, remoteness of users, lack of social cues and the increase in publicity make it difficult for a bystander to go through all 5 steps necessary to intervene in a situation [99].

### 4.5. Parents

In this section, we summarize our discussion of the response to *RQ7 What aspects of cyberbullying are underestimated by parents of adolescents*. Studies show that parents can play a crucial role in cyberbullying situations, as well as in cyber victimization and cyber perpetration [15,92,95]. Russian parents often underestimate the experience of encountering cyberbullying or find it difficult to assess such experience. Let us look at some aspects. Only one in five parents of the adolescents aged 12 to 13 and one in four of the adolescents aged 14 to 17 is aware of the use of information from the victim’s page to carry out cyberbullying. At the same time, this is one of the most common tactics of cyberbullying; half of the adolescents reported it. The reasons for this lack of awareness can be as follows: firstly, parents might not be aware of the specifics of their child’s digital socialization, including not understanding the importance of online self-presentation for adolescents and, therefore, not monitoring it. Secondly, such parents cannot teach children privacy rules within digital sociality, which can lead to self-disclosure and oversharing online. Combined, this leads to a greater risk of victimization of children and adolescents.

More than a third of the parents find it difficult to assess the strength and duration of the emotional response of their children who have become victims of cyberbullying. They also greatly underestimate the circle of acquaintances (for example, classmates) as a source of cyberbullies. The parents underestimate how many children do not turn to anyone for support and overestimate how often children turn to them for support. On the whole, the ignorance of the parents prevents the adolescents from receiving necessary social support and adopting constructive coping strategies in victimization incidents, especially in the case of the younger adolescents. This can be aggravated by the digital divide, when parents might not take interest in the online daily life of adolescents and might not have sufficient competence to provide the necessary assistance [59,64,65].

One in five parents of the younger adolescents and one in four of the older ones find it difficult to assess the behavioral strategies of cyberbullying bystanders. The parents also underestimate the prevalence of inaction, victim’s protection and aggressor’s support. Such ignorance can make it difficult for parents to form the prosocial behavior of adolescents in a cyberbullying situation and to increase the level of safety of the digital environment. Thus, another characteristic of digital sociality is the lack of adult supervision and a gap in the intergenerational transfer of experience of social interaction practices.

### 4.6. Limitations and Future Directions

The limitations of the study include the used self-report method and the possible occurrence of social desirability bias when all the three generations answer the questions about their cyberbullying experience. Thus, the results obtained regarding a portion of respondents who did not experience strong emotions or immediately recovered when faced with cyberbullying as a victim may indicate a certain distorted subjective perception of the situation. It can be assumed that despite the study’s setting to assess cyberbullying specifically, respondents may have relied on their experience of encountering different situations of cyber aggression, such as flaming or trolling, which may have less complex consequences compared to cyberbullying. It is also important to note that the results regarding bystanders are based on subjective perceptions of their behavior, which may partially correspond to actual actions in cyberbullying. The data presented are a reflection of a certain stage of the digital everyday life of Russians before the COVID-19 pandemic and the war, which also seems to be an important limitation. Nevertheless, the findings regarding the consequences for victims, motivations of aggressors, preferred strategies of bystanders, parental unawareness and so forth, are considered relevant. They reflect the socio-psychological mechanisms of cyberbullying, which have been shown to be relatively persistent and the data are also consistent with current international research. This study remains the only large-scope socio-psychological survey of cyberbullying in Russia and repeating the research on a number of issues seems promising for understanding the dynamics of the situation. As a research perspective, the need for a comprehensive study of victims, perpetrators and bystanders in different situations of cyber aggression (for instance, cyberbullying, cyberstalking, trolling or spreading hate) should be highlighted, which will allow to obtain a more holistic understanding of the destructive side of digital sociality.

## 5. Practical Implications

The revealed specifics of digital sociality—which largely determine the motives and actions of and consequences for cyberbullying aggressors, victims and bystanders—requires close attention from researchers, educators and policy makers, especially due to the rapid advancement of digital technologies that can contribute to the emergence of both new risks and new practices of mutual aid, security and development. The identified risk groups of victimization and the lack of parents’ awareness, as a reflection of the digital divide, raise concerns and are important to take into account when drawing up cyberbullying prevention programs. When working on directions of awareness-raising activities and organizing psychological support, it is necessary to take into account the revealed age specifics in the situations when victims choose whom to turn to for social support and when cyberbullying bystanders choose prosocial activity.

## 6. Conclusions

The results obtained indicate a complex combination of the characteristics of offline and online reality in adolescents’ and youth’s cyberbullying experience, which confirms the need to study the phenomenon within mixed reality. A comparative analysis of adolescents’ and youth’s experiences and their perceptions of various aspects of cyberbullying, as well as parents’ perceptions of their children’s experiences of cyberbullying contribute to understanding the socio-psychological mechanisms of this destructive phenomenon in the context of the roles of aggressor, victim and bystander.

## Figures and Tables

**Table 1:** Gender and age characteristics of the respondents from the different samples.

Samples	Number of Males (%)	Number of Females (%)	Gender Not Specified, Number of Respondents (%)	Average Age ± Standard Deviation
Adolescents aged 14 to 17	484 (47.0%)	535 (52.0%)	10 (1.0%)	15.47 ± 1.09
Adolescents aged 12 to 13	240 (45.7%)	279 (53.1%)	6 (1.1%)	12.42 ± 0.58
Youth	300 (40.8%)	436 (59.2%)	0 (0%)	23.33 ± 3.90
Parents of adolescents	214 (19.4%)	877 (79.4%)	14 (1.3%)	41.21 ± 5.63

**Table 2:** Cyberbullying initiators through the eyes of three generations.

Cyberbullying Initiators	Adolescents Aged 12 to 13	Adolescents Aged 14 to 17	Youth	Parents of Adolescents Aged 12 to 13	Parents of Adolescents Aged 14 to 17	Chi-Square (Differences among 5 Groups)	Cramer’s V (Differences among 5 Groups)
Classmates	68.2%	68.1%	38.7%	42.9%	49.3%	92.2 **	0.27
Students from the same year level	38.2%	33.2%	6.6%	19.6%	15.0%	106.36 **	0.28
Anonymous users	31.9%	28.1%	33.1%	19.6%	28.8%	9.3	0.08
Friends	25.7%	27.3%	27.2%	9.4%	6.0%	67.95 **	0.22
High school students	20.9%	19.3%	0.3%	6.5%	6.7%	88.23 **	0.25
Acquaintances	20.0%	37.1%	39.7%	18.8%	18.0%	60.48 **	0.21
Internet friends	16.2%	24.0%	33.1%	13.8%	29.2%	30.06 **	0.15
Teachers	4.2%	5.5%	0.3%	0.0%	1.9%	23.91 **	0.13
Parents	1.6%	1.4%	0.3%	0.0%	0.7%	4.52	0.06
Siblings	1.6%	3.1%	0.7%	0.0%	1.1%	10.21 *	0.09
I find it difficult to answer	-	-	-	25.4%	17.2%	-	-

*—*p* < 0.05, **—*p* < 0.01.

**Table 3:** Aggressive acts in cyberbullying: comparing the responses of the adolescents, youth and parents.

Acts towards a Victim in a Cyberbullying Situation	Adolescents Aged 14 to 17	Youth	Parents of Adolescents Aged 12 to 13	Parents of Adolescents Aged 14 to 17	Chi-Square (Differences among 4 Groups)	Cramer’s V (Differences among 4 Groups)
Was excluded or deleted from a group chat or community	55.8%	47.9%	39.1%	40.6%	22.03 **	0.14
Was unfriended	47.8%	45.7%	50.7%	48.1%	0.96	0.03
Personal data, photos and videos from the victim’s personal page were used against him or her	47.6%	47.5%	18.8%	27.1%	62.78 **	0.23
False information was posted about the victim	43.9%	52.1%	25.4%	35.0%	33.72 **	0.17
Rude and unpleasant polls about the victim were created	35.6%	25.5%	14.5%	16.5%	44.47 **	0.20
Groups, communities or pages were created on social media, where offensive information was posted about the victim	22.7%	29.1%	15.2%	16.5%	16.70 **	0.12
Personal data (first and last name, photos, etc.) from an online profile were used to create a fake account	18.5%	24.1%	8.0%	16.5%	16.87 **	0.12
The password from the victim’s account was stolen in order to publish or send negative and inappropriate information on his or her behalf	9.1%	16.7%	13.0%	10.2%	10.75 *	0.10
Insulting and humiliating information about the victim was sent to the friends, parents and teachers	15.9%	19.1%	13.0%	12.0%	6.02	0.07

*—*p* < 0.05, **—*p* < 0.01.

**Table 4:** Seeking help in a cyberbullying situation.

To Whom the Respondents Turn to for Help in a Cyberbullying Situation	Adolescents Aged 12 to 13	Adolescents Aged 14 to 17	Youth	Parents of the Adolescents Aged 12 to 13	Parents of the Adolescents Aged 14 to 17	Chi-Square (Differences among 5 Groups)	Cramer’s V (Differences among 5 Groups)
Parents	38.5%	26.6%	16.3%	42.1%	34.0%	52.82 **	0.18
Friends	36.9%	45.8	24.1%	11.0%	12.3%	145.67 **	0.30
No one	26.0%	25.8%	32.2%	4.1%	8.7%	83.93 **	0.23
Sibling	19.6%	14.3%	4.4%	11.0%	6.0%	47.23 **	0.17
Teacher	11.9%	3.9%	1.4%	6.2%	2.3%	46.32 **	0.17
Trusted adult	9.6%	6.4%	6.1%	3.4%	3.0%	13.69 **	0.09
Law enforcement agencies (police)	7.4%	2.9%	2.0%	1.4%	2.0%	21.38 **	0.12
Specialized services (psychologist, social worker, etc.)	4.5%	4.3%	3.1%	0.7%	1.0%	11.58 *	0.09
I find it difficult to answer	-	-	-	16.6%	17.7%	-	-

*—*p* < 0.05, **—*p* < 0.01.

## Data Availability

The data presented in this study are available upon request from the corresponding author. The data are not publicly available due to privacy and ethical restrictions.
